# Psychological and immunological associations with movement-evoked low back pain among older adults

**DOI:** 10.1097/PR9.0000000000001262

**Published:** 2025-04-03

**Authors:** Riley Kahan, Arthur Woznowski-Vu, Janet L. Huebner, Carl F. Pieper, Adam P. Goode, Steven Z. George, Timothy H. Wideman, Virginia Byers Kraus, Cathleen Colón-Emeric, Corey B. Simon

**Affiliations:** a Department of Surgery, Duke University School of Medicine, Durham, NC, USA; b School of Physical & Occupational Therapy, McGill University, Montreal, QC, Canada; c Duke Molecular Physiology Institute, Duke University School of Medicine, Durham, NC, USA; d Center for Aging and Human Development, Duke University School of Medicine, Durham, NC, USA; e Department of Biostatistics and Bioinformatics, Duke University School of Medicine, Durham, NC, USA; f Department of Orthopaedic Surgery, Duke University School of Medicine, Durham, NC, USA; g Duke Clinical Research Institute, Durham, NC, USA; h Department of Population Health Sciences, Duke University School of Medicine, Durham, NC, USA; i Department of Medicine, Duke University School of Medicine, Durham, NC, USA; j Durham VA Geriatric Research Education and Clinical Center, Durham, NC, USA

**Keywords:** Older adults, Low back pain, Movement-evoked, Catastrophizing, Inflammation, Reactivity

## Abstract

**Introduction:**

Low back pain (LBP) is a leading global factor in disability among older adults. Movement-evoked pain (MEP) is potentially an important mediator in the disability pathway but is predominantly tested in the laboratory.

**Objectives:**

We aimed to explore MEP in the natural environment (“daily” MEP) and its correlation with laboratory MEP, along with potential psychological and immunological influences.

**Method:**

Thirty-five older adults with persistent LBP attended a single laboratory session. Pain catastrophizing, pain-related fear of movement, and pain self-efficacy were measured by questionnaire. Resting inflammation and inflammatory reactivity to painful movement were evaluated using serum interleukin-6, tissue necrosis factor alpha, and C-reactive protein (CRP). Laboratory MEP was defined by aggregate pain intensity with a movement provocation test. Daily MEP was measured for the next 7 days using ecological momentary assessment.

**Results:**

Laboratory MEP was strongly correlated with daily MEP (*ρ* = 0.780, *P* = <0.001). C-reactive protein (Hedges [*g*] = 0.266) and interleukin-6 (g = 0.433) demonstrated small to moderate reactivity to painful movement. After controlling for age and multimorbidity, pain catastrophizing and pain self-efficacy explained 24% to 37% variance in laboratory and daily MEP. Resting inflammatory markers were not associated with MEP; however, C-reactive protein reactivity to painful movement explained 19% to 25% variance in laboratory and daily MEP.

**Conclusion:**

Preliminary indication is that laboratory and daily MEP may be proxy measures for one another, and that MEP is influenced by psychological and immunological factors. Future studies will aim to (1) validate findings among older adults with persistent LBP and (2) for clinical phenotyping, clarify complex relationships among psychological and immunological factors with disability pathway components like MEP.

## Abstract 2

Supplemental Digital Content is Available in the Text.

In older adults with low back pain, laboratory and daily movement-evoked pain (MEP) were strongly correlated; psychological and immunological factors explained variance in both MEP outcomes.

## 1. Introduction

Globally, low back pain (LBP) is the most disabling pain condition among older adults and a risk factor for disability, falls, morbidity, and mortality.^6,9,13,31–33,61^ Pathways from geriatric LBP to negative health outcomes are poorly understood, although a potential component of such pathways is movement-evoked pain (MEP).^7^ Movement-evoked pain is pain experienced with gross movements like bending and walking. Movement-evoked pain occurs in over 80% of older adults with LBP^21^; accumulating research links MEP with worse physical function and disability than LBP at rest.^21,22,24,51^

However, the utility of MEP in clinical assessment remains limited by multiple knowledge gaps. First, most studies to date assessed MEP in laboratory settings rather than during daily tasks in the natural environment (ie, “daily” MEP).^7,11^ In particular, it is unknown whether laboratory MEP—eg, pain evoked with standardized movement-based provocation tests^51^—is a valid proxy measure of daily MEP. Second, we lack knowledge of the mechanistic factors underlying MEP, most notably the role of psychological distress. Three psychological factors consistently predict disability and musculoskeletal pain at rest: pain catastrophizing,^57^ pain-related fear of movement,^36^ and pain self-efficacy.^37^ However, to our knowledge, only 4 studies have tested these factors against MEP in older adults with LBP.^22,24,39,44^ All 4 studies employed laboratory MEP (ie, not daily MEP), and only 1 of the 3 psychological factors—pain self-efficacy—was uniformly associated with MEP across studies.^22,24,39,44^

Third, there is a lack of evidence on the relationship between MEP and immunological factors, which are known to mediate nociception and pathological pain.^8,19,45,62^ Previous literature has identified 3 inflammatory markers that were higher when compared to healthy controls or were associated with LBP intensity: interleukin-6 (IL-6), tissue necrosis factor alpha (TNF-⍺), and C-reactive protein (CRP).^3,16,28,40^ However, only a handful of previous studies included older adults^18,30,47–49,63^; no study, to our knowledge, assessed the relationship of inflammatory markers to MEP in older adults. Moreover, previous studies mostly tested resting inflammation and not inflammatory *change* after painful stimuli (also known as “reactivity”)^34,35^; though a recent case-control analysis found IL-6 reactivity was associated with MEP in younger and middle-aged adults with LBP.^64^ It is important to examine inflammatory reactivity in older adults due to previous findings of dysregulation, such that circulating inflammation increased substantially with experimentally induced pain.^12,26,50^ To this end, our working hypothesis is that older adults experience a similar inflammatory reactivity to painful movement.

In this study, we sought to address the aforementioned knowledge gaps in a sample of older adults with persistent LBP. Our first aim was to test associations between daily MEP measured via 7-day ecological momentary assessment (EMA), and laboratory MEP after the MEPLO test (“Movement-Evoked Provocation Test of Low Back Pain in Older Adults”).^51^ Our second aim was to quantify inflammatory reactivity to painful movement in IL-6, TNF-⍺, and CRP, which were selected based on the aformentioned resting inflammation and experimental pain reactivity studies. Finally, an exploratory aim was to test associations between psychological factors, immunological factors, and MEP. The 2 overarching goals of this study were to (1) describe the generalizability between MEP laboratory measures and daily MEP measures and (2) inform future research into the underlying biopsychosocial mechanisms of the MEP-disability pathway. Completion of these goals will expedite clinical phenotyping of—and treatment tailoring for—older adults with LBP who are high risk for MEP and subsequent disability.

## 2. Methods

### 2.1. Brief summary of study procedures

**Figure 1.**
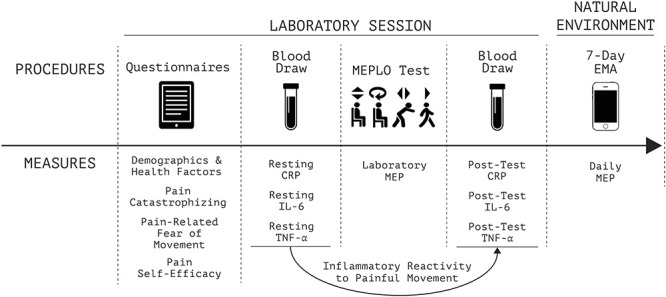
Study procedures and measures. EMA, ecological momentary assessment; MEPLO, movement-evoked provocation test for low back pain in older adults.^51^

Study procedures and measures are illustrated in Figure 1. The study consisted of 1 laboratory session followed by 7 days in the natural environment. After consent and screening, participants provided demographic, health, and psychological information by questionnaire. Next, a study nurse collected a whole blood sample. Participants then performed the MEPLO test to quantify laboratory MEP, after which (3 minutes post-test) a study nurse collected a second whole blood sample. Finally, participants completed EMA via smartphone application for 7 days to define daily MEP. This study was approved by the Duke University Institutional Review Board.^51^

### 2.2. Participants

Forty community-ambulating older adults with persistent LBP, recruited from 2 clinical research databases, were enrolled. Eligible participants were between 60 and 85 years of age, experienced LBP for at least 3 months, reported LBP as their primary pain complaint, and reported average daily LBP pain intensity of at least 40/100 on a 0 to 100 numeric pain rating scale (NPRS; 100 = worst pain imaginable).

Exclusion criteria were as follows: (1) LBP from trauma (eg, work accident, car accident, fall); (2) previous surgery for LBP; (3) changes in LBP treatment in the past month; (4) current daily use of opioids; (5) current active malignancy (except skin); (6) rheumatic disease other than osteoarthritis (eg, rheumatic arthritis, systemic lupus erythematosus, fibromyalgia); and (7) psychiatric-related hospital admission in the past year. An on-site global cognitive screen was performed using the Montreal Cognitive Assessment^41^; those scoring less than 21 were excluded from the study.^38^ Vital signs were also screened, with systolic blood pressure >170, diastolic blood pressure >90, or resting heart rate >100 resulting in study exclusion. Finally, to prioritize safety with the MEPLO test, participants received a neurological screen (light touch and vibratory sense) to confirm adequate sensation in their feet; participants who incorrectly reported sensation 3 out of 5 times on either foot were excluded.

### 2.3. Study measures

#### 2.3.1. Demographic risk factors

Age, sex, race, LBP duration, and multimorbidity were collected by self-report for descriptive purposes, and in the case of age and multimorbidity, as covariates. To reduce the number of model covariates, we created a multimorbidity index score based on the number of the following medical conditions: hypertension, diabetes, heart disease, pulmonary disease, skin cancer, peripheral vascular disease, and stroke/neurological disease.

#### 2.3.2. Psychological factors

Pain catastrophizing is an exaggerated appraisal of future events and the expectation of a worse pain experience^46^; it was measured using the Pain Catastrophizing Scale (PCS).^43,55^ Each of the 13 items is rated on 5-point Likert scale (0 = “not at all”; 4 = “all the time”); scores range from 0 to 52, with higher scores reflecting higher pain catastrophizing. Pain-related fear of movement is the emotional reaction to movement perceived as threatening^36^; it was measured using the Tampa Scale for Kinesiophobia Short Form (TSK-11).^59^ Participants rate items on a 4-point Likert scale (1 = “strongly disagree”; 4 = “strongly agree”); scores range from 11 to 44, with higher scores reflecting higher pain-related fear of movement. Finally, pain self-efficacy is belief in one's ability to manage pain^2^; it was measured using the Self-efficacy for Pain Management Subscale of the Chronic Pain Self-Efficacy Scale.^1,29^ Participants rate 5 questions using 0 to 10 visual analog scale (0 = “very uncertain”; 10 = “very certain”). Since other scales in the study use a 0 anchor, we modified the Chronic Pain Self-Efficacy Scale from the original 10 to 100 to 0 to 100 to avoid confusion.

#### 2.3.3. Blood collection preparation and processing

Before the session, participants were asked to refrain from caffeine, alcohol, and exercise 12 hours before arriving and to fast for at least 2 hours before arriving. Sessions were scheduled between mid-morning and early afternoon. To limit physical activity before testing, participants were transported from their vehicle to the laboratory by wheelchair. Participants were in a resting, seated position for at least 30 minutes before the first blood draw. A registered study nurse then collected approximately 5 mL of blood in a serum separator tube (SST) before and 3 minutes after the MEPLO test. Blood was transferred to the processing lab and after 30 minutes of clotting time was spun in a centrifuge at 3000 rpm for 15 minutes. Serum was then divided into aliquots and stored in a −80°C freezer. At the completion of the study, samples were transported on dry ice to the Duke Biomarker Core Facility for batched analysis.

#### 2.3.4. Immunological factors

##### 2.3.4.1. Quantifying C-reactive protein, interleukin-6, and tissue necrosis factor alpha

Concentrations of CRP, IL-6, and TNF-α were quantified by sandwich immunoassays using electrochemiluminescent detection (Meso Scale Diagnostics, Rockville, MD). Assay precision and detection rates are detailed in the Supplemental Table, http://links.lww.com/PR9/A295. A human control serum sample was provided by the Duke Biomarker Core Facility and run in duplicate on every plate to assess variability; as well as to establish a control range for each assay. Control samples were within the acceptable control range for all plates and analytes. Intra- and interassay coefficients of variation for the 3 markers were between 2.8% and 7%. Over 99% of assays were within the quantitative range.

##### 2.3.4.2. Inflammatory reactivity to painful movement

Inflammatory reactivity is the multipoint measure of immune response to a physiologic stressor.^12,14,20,25,50^ In such paradigms, participants act as their own control.^17^ Previous work showed that older adults display pronounced inflammatory reactivity starting 15 minutes after a pain stressor.^12^ The MEPLO test takes approximately 18 minutes to complete,^51^ and duration between blood draws was a mean 21 minutes (SD 2.8 minutes).

To quantify inflammatory reactivity to painful movement, values of each biomarker at rest and after the MEPLO test were log-transformed to address concentration skewness. Log-transformed values were then entered into a within-subject statistical test of difference (ie, paired *t*-test). For tests of association (eg, bivariate correlations), inflammatory reactivity to painful movement was calculated using 2 methods. First, change in inflammatory reactivity to painful movement was quantified by subtracting log-transformed post-test values from log-transformed resting values; post-test—resting difference in log-transformed values equates to a normalized ratio (log [post/pre]). Second, normalized percent change was quantified by subtracting raw resting values from raw post-test values, dividing the difference by the raw resting value, and z-transforming the final number. We used 2 calculations to account for equation variability across research fields, to examine the stability of models, and to inform future work.

#### 2.3.5. Pain measures

##### 2.3.5.1. Laboratory movement-evoked pain

The MEPLO test is fully described elsewhere,^51^ but briefly, participants performed 4 standardized movements: repetitive chair rises, repetitive trunk rotation, repetitive standing forward reach, and a 6-minute walk. Using a 0 to 100 Numeric Pain Rating Scale (NPRS; 0 = “no pain”; 100 = “worst pain imaginable”), participants reported their pain at 5 time points: before the MEPLO test (pretest resting pain), and after each of the 4 included movements. Laboratory MEP was calculated by adding the 4 pain ratings immediately after the 4 movements (0–100 NPRS), and subtracting pretest resting pain (0–100 NPRS). Notably, aggregate calculation of MEP is believed to be superior to simple delta calculation (ie, post- minus pretest pain), based on associations with physical function and disability outcomes in older adults with LBP.^22,51^

##### 2.3.5.2. Daily movement-evoked pain

Daily MEP was collected using an EMA smartphone application (RealLife EXP smartphone application, LifeData, LLC. Marion, IN). Research staff assisted in downloading and educating participants in the smartphone application. For up to 7 days, participants were notified twice a day (12 pm and 8 pm) to answer survey questions about MEP experienced in the morning and evening. Participant data were automatically uploaded to an encrypted cloud-based storage platform. Once downloaded, data were cleaned and organized by 2 study authors (R.K. and A.W.). Daily MEP was calculated by averaging the worst pain (0–100 NPRS) experienced during the movement or physical task considered most painful for each time period.

### 2.4. Statistical analysis

Analyses were completed using IBM SPSS Statistics software, Version 29 (2022; IBM Corp., Armonk, NY). Descriptive statistics were performed for all factors. Ecological momentary assessment completion rates, patterns of missing data (eg, Little's Missing Completely at Random [MCAR] test), and associated risk of bias were characterized across the variables of interest. Most participants (83%) completed between 90% and 100% of individual EMA entries, while 91.5% had at least an 80% completion rate. The average EMA completion rate for daily MEP was 90.4% (SD = 11.7%). Excluding data related to EMA entries, only 1.30% of total data were missing and all pertained to immunological factors—5 participants were lacking data due to unsuccessful blood draws. Moreover, Little's MCAR test supports the pattern of missing data as MCAR (*P* = 0.200). Therefore, data appeared to be MCAR with a relatively low proportion of missing data. The samples with all data (n = 30) vs those missing immunological data (n = 5; via independent *t*-tests) did not show any differences (*P* > 0.05). Given the low risk of bias due to missing data, we proceeded with a complete case analysis for each of the analyses.

Repeated Measures ANOVA tested differences in daily MEP across days. Paired t-tests were used to test differences in log-transformed inflammatory marker values at rest and after the MEPLO test (ie, inflammatory reactivity to painful movement); one-tailed *P*-value was used based on the anticipated marker increase for older adults experiencing a painful stressor.^12,50^ Inflammatory reactivity effect sizes were approximated from Hedges *g* estimates recommended for interpreting data from older adults^5^: *g* = 0.15 small (weak), 0.40 moderate, and 0.75 large (strong). Spearman rank-order correlation analysis (rho) was used to examine bivariate associations among psychological factors, immunological factors (both normalized ratio and percent change scores), and laboratory and daily MEP. We opted for rho as it is robust to outliers (versus Pearson r, which is sensitive to outliers) and is more likely to underestimate population values in small sample sizes.^58^ To categorize association effect sizes, we used benchmarks recommended for interpreting data from older adults^5^: = 0.10 small (weak), 0.20 medium, and 0.30 large (strong). Finally, multivariable ordinary least squares (OLS) regression models were constructed to assess the unique contribution of psychological and immunological factors to the variance in MEP, after (1) accounting for age and multimorbidity and (2) comparing the strongest psychological and immunological factors in the same model. Given the exploratory nature of these analyses, multiple test correction was not performed. As an alternative to correcting for multiple tests and to monitor model stability and multicollinearity, we set a priori cutoffs for intercorrelation (r < 0.70), tolerance (r > 0.20), and variance inflation (r < 4).

## 3. Results

Thirty-nine of the 40 enrolled participants passed all screens. Thirty-five participants provided laboratory MEP, daily MEP, and psychological data. Of this sample, 66% identified as White, 31% Black, and 3% Asian. Approximately one-third (29%) had a high school degree or completed some college work; 42% had a college degree or completed some postgraduate work; and 29% had a postgraduate degree. The mean age for the sample was 67 years, and 54% identified as female. Of the 39 participants enrolled, 30 participants had completed EMA to measure daily MEP, and successful blood draws to measure resting inflammatory markers and inflammatory reactivity. Participant and study factors are further described in Table 1.

**Table 1:** Descriptive data for demographic, pain, psychological, and immunological factors.

	Range (min–max)	Mean (SD)	Median or %
Demographic & health factors			
Age (y)	60 to 76	66.9 (4.6)	66
Biological sex (female, n)		19	54%
LBP duration (y)	1 to 55	13.9 (14.9)	7.0
Multimorbidity index*	0 to 4	0.9 (1.0)	1.0
Pain factors			
Laboratory MEP (Aggregate)†	−15.0 to 230.0	106.5 (58.2)	105.0
Daily MEP (0–100)‡	5.3 to 72.7	36.7 (17.9)	33.6
Psychological factors			
Pain catastrophizing (0–52)	0 to 29	10.5 (7.2)	10.0
Pain-related fear of movement (11–44)	14 to 36	23.7 (17.4)	24.0
Pain self-efficacy (0–100)	34 to 100	71.2 (17.4)	74.0
Immunological factors			
Resting CRP (ng/mL)	118 to 17265	2992 (3410)	1702
Resting IL-6 (pg/mL)	0.24 to 4.77	0.99 (0.85)	0.83
Resting TNF-⍺ (pg/mL)	0.83 to 5.33	2.77 (0.80)	2.70

* Medical conditions coded by presence or absence (yes = 1, no = 0), summated to create a weighted Multimorbidity Index score.

† Aggregate score of 4 pain ratings immediately after the 4 movements in the MEPLO test, minus pretest resting pain.

‡ Average daily MEP for all events.

CRP, C-reactive protein; IL-6, interleukin-6; LBP, low back pain; MEP, movement-evoked pain; MEPLO, movement-evoked provocation test for low back pain in older adults; TNF-⍺, tissue necrosis factor alpha.

**Figure 2.**
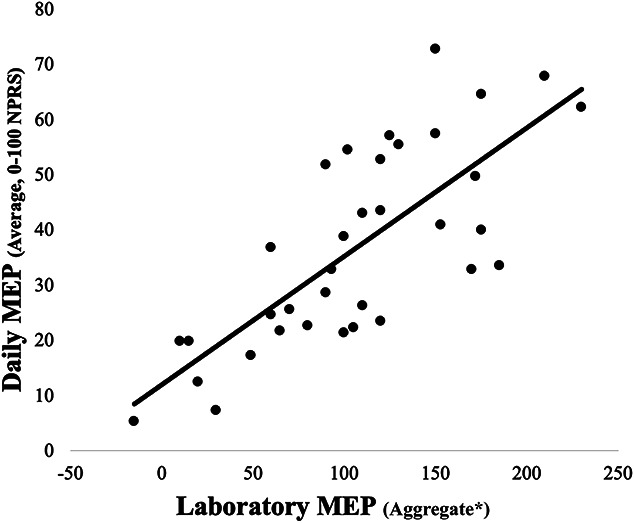
Association between laboratory MEP (MEPLO test) and daily MEP (7-day EMA). EMA, ecological momentary assessment; MEPLO, movement-evoked provocation test for low back pain in older adults. (*) Aggregate score of 4 pain ratings immediately after the 4 movements in the MEPLO test, minus pretest resting pain.

Daily MEP ranged from 33.8 to 39.8 across 7 days (mean = 36.7, average SD = 17.9) and did not significantly change (*P* = 0.630). A strong, positive association was observed between laboratory MEP and daily MEP (*ρ* = 0.780, *P* = <0.001; Fig. 2).

Tests of inflammatory reactivity to painful movement are described in Table 2 and illustrated in Supplemental Figure 1, http://links.lww.com/PR9/A295. Across all participants pre- to post-MEPLO test, a small increase in CRP and a moderate increase in IL-6 occurred. Tissue necrosis factor alpha did not appear to change.

**Table 2:** Inflammatory reactivity to painful movement (resting to postmovement-evoked provocation test for low back pain in older adults test difference).

Immunological factors	Resting	Post-MEPLO test	*P* (difference)	Hedges G
	Mean (SD)	Mean (SD)		
CRP (log-transformed)	3.25 (0.48)	3.27 (0.50)	0.074	0.266
IL-6 (log-transformed)	−0.09 (0.26)	−0.05 (0.26)	0.011	0.433
TNF-α (log-transformed)	0.42 (0.14)	0.43 (0.12)	0.301	0.096

CRP, C-reactive protein; IL-6, interleukin-6; MEPLO, movement-evoked provocation test for low back pain in older adults; TNF-⍺, tissue necrosis factor alpha.

Bivariate associations are presented in Table 3. Higher pain catastrophizing and lower pain self-efficacy were strongly associated with higher laboratory MEP and daily MEP (Supplemental Figure 2A,B, http://links.lww.com/PR9/A295). Resting inflammation values did not appear to correlate. Ratio and percent change CRP reactivity to painful movement were moderately and positively associated with both laboratory MEP and MEP (Supplemental Figure 2C, http://links.lww.com/PR9/A295). Interleukin-6 and TNF-⍺ reactivity to painful movement measures were not associated with either laboratory MEP or daily MEP.

**Table 3:** Bivariate associations with movement-evoked pain.

Factors	Laboratory MEP		Daily MEP	
	Rho (ρ)	*P*	Rho (ρ)	*P*
Psychological factors				
Pain catastrophizing	**0.551**	**<0.001**	**0.607**	**<0.001**
Pain-related fear of movement	0.275	0.110	0.280	0.103
Pain self-efficacy	**−0.518**	**<0.001**	**−0.519**	**<0.001**
Immunological factors				
CRP				
Resting (log-transformed)	0.159	0.401	0.308	0.098
Reactivity to painful movement—Ratio*	**0.375**	**0.041**	**0.426**	**0.019**
Reactivity to painful movement—Percent change†	**0.363**	**0.049**	**0.417**	**0.022**
IL-6				
Resting (log-transformed)	0.001	0.997	0.093	0.626
Reactivity to painful movement—Ratio*	−0.061	0.748	−0.084	0.659
Reactivity to painful movement—Percent change†	−0.001	0.994	−0.052	0.786
TNF-α				
Resting (log-transformed)	−0.036	0.852	−0.127	0.502
Reactivity to painful movement—Ratio*	0.119	0.531	0.033	0.862
Reactivity to painful movement—Percent change†	0.119	0.531	0.033	0.862

Bold = *P* < 0.05.

* Post–pretest difference in log-transformed values, equating to a normalized ratio.

† Nonlog-transformed, z-score normalized percent change score.

CRP, C-reactive protein; IL-6, interleukin-6; MEP, movement-evoked pain; TNF-⍺, tissue necrosis factor alpha.

Multivariable associations accounting for age and multimorbidity are presented in Table 4A. Age and multimorbidity accounted for 1% and 4% variance in laboratory and daily MEP. In laboratory MEP and daily MEP models: pain catastrophizing explained 36% and 37% variance, pain self-efficacy 24% variance, ratio CRP reactivity to painful movement 19% and 22% variance, and percent change CRP reactivity to painful movement 23% and 25% variance.

**Table 4:** Multivariable associations with movement-evoked pain.

A) Controlling for age and multimorbidity (separate models)				
Model	Factor	R^2^∆	Beta	Omnibus F ∆
Dependent variable: Laboratory MEP				
1	Pain catastrophizing	0.36	0.638	F_1,30_ = 17.1, *P* = <0.001
2	Pain-related fear of movement	0.10	0.318	F_1,30_ = 3.3, *P* = 0.080
3	Pain self-efficacy	0.24	−0.59	F_1,30_ = 9.4, *P* = 0.005
4	CRP reactivity to painful movement—Ratio*	0.19	0.445	F_1,26_ = 6.3, *P* = 0.018
5	CRP reactivity to painful movement—Percent change†	0.23	0.491	F_1,26_ = 8.1, *P* = 0.009
Dependent variable: Daily MEP				
1	Pain catastrophizing	0.37	0.649	F_1,30_ = 19.2, *P* = <0.001
2	Pain-related fear of movement	0.09	0.311	F_1,30_ = 3.3, *P* = 0.081
3	Pain self-efficacy	0.24	−0.595	F_1,30_ = 10.1, *P* = 0.003
4	CRP reactivity to painful movement—Ratio*	0.22	0.476	F_1,26_ = 7.9, *P* = 0.009
5	CRP reactivity to painful movement—Percent change†	0.25	0.502	F_1,26_ = 9.0, *P* = 0.006

B) Final model, strongest factors				
Factor	R2/R^2^∆	Beta	*P*	Omnibus F, final model
Dependent variable: Laboratory MEP				
Pain catastrophizing	0.29	0.328	0.127	F_3,29_ = 6.09, *P* = 0.003
Pain self-efficacy	0.02	−0.169	0.412	
CRP reactivity to painful movement—Percent change†	0.10	0.327	0.049	
Dependent variable: Daily MEP				
Pain catastrophizing	0.43	0.529	0.011	F_3,29_ = 8.61, *P* = <0.001
Pain self-efficacy	0.00	−0.056	0.768	
CRP reactivity to painful movement—Percent change†	0.07	0.278	0.068	

* Post–pretest difference in log-transformed values, equating to a normalized ratio.

† Nonlog-transformed, z-score normalized percent change score.

CRP, C-reactive protein; MEP, movement-evoked pain.

Finally, multivariable associations accounting for the strongest psychological and immunological factors were explored (Table 4B). Pain catastrophizing, pain self-efficacy, and percent change CRP reactivity to painful movement accounted for 41% total variance in laboratory MEP. Similarly, pain catastrophizing, pain self-efficacy, and percent change CRP reactivity to painful movement accounted for 50% total variance in daily MEP.

## 4. Discussion

Low back pain has a high global impact on older adults,^6,9^ but not all older adults with LBP succumb to disability.^52^ Moreover, the pathway from geriatric LBP to negative health outcomes—eg, falls, disability, morbidity, and mortality—is poorly understood. Until pathway components and processes are identified, clinicians will continue having difficulty in preventing such negative health outcomes in high-risk patients. This study addresses knowledge gaps for a seemingly integral pathway component among older adults with LBP—MEP.^21,22,24,51^ Particularly notable was the strong correlation between laboratory MEP and daily MEP, which suggests that these 2 measures may be interchangeable and/or proxy for one another in future study.

This study also indicated a strong relationship between MEP (laboratory and daily) and psychological factors, which are well known predictors of musculoskeletal pain, and yet, mostly studied in relation to resting pain and recalled pain (ie, pain days or weeks prior).^36,37,57^ Importantly, MEP is believed to be more severe than resting pain,^54^ and since its stimulus (movement) is integral to daily living, the underlying psychology of MEP might differ from resting or recalled pain. Findings corroborate previous geriatric LBP studies that found associations between pain self-efficacy and MEP.^22,24,39,44^ Findings also align with a recent study in middle-aged adults with LBP, in whom pain catastrophizing to painful movement influenced daily pain and mood.^60^ At the same time, recent prospective geriatric LBP studies have shown that MEP predicts 1 year disability but psychological distress does not.^23,24^ Also, despite its specificity, pain-related fear of movement demonstrated the weakest association with MEP of the 3 psychological factors. Therefore, future work is needed to clarify the complex temporal ordering (including but not limited to fluctuations over time) of psychological factors in relation to MEP and disability. Future studies must also qualitatively assess patient and clinician partners to better understand psychological factors in the context of MEP experience and the potential to enhance geriatric LBP management. Such work is a priority, given the current clinical misconceptions surrounding pain-related psychological distress factors like pain catastrophizing.^56^

In quantifying inflammatory reactivity across the entire sample, we observed a small and moderate increase for CRP and IL-6, respectively. The first study, to our knowledge, to quantify inflammatory reactivity in response to painful movement was by Sowa et al.^53^ in older adults with LBP. Group aggregates for inflammatory reactivity were not reported, although CCL5 increased for most of the sample performing the Short Physical Performance Battery (SPPB). A recent LBP case-control study by Flegg et al.^64^ did not find IL-6 to change with painful movement; notably, however, participants were younger and middle-aged. Looser comparisons can be made with studies using experimental pain stressors (eg, noxious heat and cold) instead of painful movement.^12,26,50^ Lee et al.^26^ reported a modest increase in IL-6, but not CRP or TNF, with experimental pain evocation among late middle-aged adults with knee osteoarthritis. Comparisons by age group have shown that pain-free older adults have more pronounced inflammatory reactivity to experimental pain than pain-free younger adults, suggesting senescent changes to immune involvement in pain.^12,50^ Like Lee et al., group aggregate inflammatory reactivity here was relatively modest; however, we note that responses were quite variable such that high reactivity occurred in some, while low to no reactivity occurred in others. A similar observation was made for CCL5 in the study by Sowa et al.^53^ Accordingly, group aggregates are likely influenced by disordinal changes cancelling out. That said, such phenomena are helpful in identifying high- or low-risk clinical phenotypes, provided associations exist with clinical outcomes like MEP and physical function, which, in older adults with LBP, are also highly variable.^31,52^

Contrary to previous LBP studies,^3,16,28,40^ this study did not find an association between resting inflammatory markers and pain outcomes, but again, previous work was specific to resting/recalled pain, not MEP. Plausibly, the dynamic nature of MEP may relate to inflammatory changes more than markers at rest. This study may be one of only two to test associations between inflammatory reactivity to painful movement and MEP ratings. Where Flegg et al. found associations between IL-6 and MEP in younger and middle-aged adults with LBP,^64^ we did not find a similar association for older adults with LBP. However, positive associations between CRP reactivity and MEP corroborate Lee et al. finding among late middle-aged adults with osteoarthritis;^26^ post-test CRP positively correlated with experimental pain sensitivity (notably, the group aggregate for CRP reactivity did not change). For older adults with LBP, Sowa et al.^53^ did not measure MEP specifically but found CCL5 reactivity to the SPPB was associated with worse SPPB performance and McGill Pain Questionnaire scores. Collectively, these 4 studies suggest that inflammatory reactivity is indeed associated with painful stressors, but may be dependent upon age, pain condition, or stressor type. For the purposes of high-risk clinical phenotyping and treatment targeting, extensive work remains to untangle the complex, bidirectional relationships between pain and the immune system. The complexity of such work necessitates adoption of innovative research frameworks, like those proposed in the fields of interoception^10,42^ and psychoneuroimmunology.^4^

Study strengths and limitations should be considered when interpreting results. Study strengths included a representative sample of older adults with persistent LBP, investigating a historically understudied pain population, measuring both negative and positive psychological factors, measuring inflammatory markers attributable to LBP, measuring both laboratory MEP and daily MEP, and using EMA to collect daily MEP. In addition, we calculated inflammatory reactivity 2 different ways and observed similar coefficients; this lends credence to the stability of associations observed with MEP and potential versatility of calculations in future studies. Regarding study limitations, this was a small pilot study intended to demonstrate feasibility; results were not adjusted for multiple comparisons and should, therefore, be considered exploratory and/or hypothesis generating. Body mass index was not accounted for although we did account for multimorbidity, which is strongly associated with obesity.^27^ Still, obesity is a well-known risk factor for higher circulating inflammation^15^ and, therefore, should be explicitly accounted for in future studies. Evidence supporting the ecologic validity of laboratory MEP should be weighed against the small sample size and validated in larger samples, as well as in comparison to a healthy control group. We chose the post-test blood draw time based on the initiation of inflammatory reactivity in experimental pain studies,^12,50^ and it is possible that longer durations may have yielded higher or lower reactivity. Notably, however is that the MEPLO test is a robust, more clinically meaningful pain stimulus when compared to experimental pain. Although we included a representative sample, we did not examine findings by race or gender identity. Daily MEP was limited to a week-long assessment, and most painful movements reported were limited to the categories provided. Finally, although EMA occurred twice a day, it was not immediately after performing painful movement.

To summarize, study findings suggest that laboratory MEP (via the MEPLO test) and daily MEP (via EMA) may be valid proxy measures for each other among older adults with LBP. Older adults with LBP demonstrated small to moderate inflammatory reactivity to painful movement as a group, with higher reactivity being associated with higher laboratory and daily MEP. Similarly, the weight and direction of psychological associations were similar across the 2 different MEP measures. To advance this work towards high-risk clinical phenotyping and subsequent tailored pain management development, future studies must (1) use innovative complex systems frameworks (eg, interoception, psychoneuroimmunology) to untangle complex relationships among psychological factors and immunological factors with disability pathway components like MEP and (2) eventually determine the acceptability, scalability, and added value of these factors for clinical management of geriatric LBP.

## Disclosures

The authors declare no conflicts of interest with this study.

## Appendix A. Supplemental digital content

Supplemental digital content associated with this article can be found online at http://links.lww.com/PR9/A295.

## Supplementary Material

SUPPLEMENTARY MATERIAL
