# Supplementary material for: Psychological and immunological associations with movement-evoked low back pain among older adults
Source: Pain Rep. 2025 Apr 3;10(3):e1262. doi: 10.1097/PR9.0000000000001262 (PMC11970892; doi:10.1097/PR9.0000000000001262)
Supplement: SUPPLEMENTARY MATERIAL [file painreports-10-e1262-s001.pdf]

Supplemental Table. Inflammatory Marker Assay Description, Precision, and Detection Rates

	**CRP**	**IL-6**	**TNF-α**
Units	ng/ml	pg/ml	pg/ml
Manufacturer	MSD (K151STD)	MSD (K151A9H)	MSD (K151A9H)
Required Volume (µl)	10	25	25
Dilution (-fold)	1000	2	2
Intra-assay CVs (within plate, %)	2.8	7.0	4.1
Inter-assay CVs (between plates, %)	3.1	6.2	6.4
Mean Actual/Reported LLOD (pg/ml)	2.04	0.06	0.06
LLOQ (pg/ml)	11.8	0.633	0.690
ULOQ (pg/ml)	184,000	488	248
Within Quantitative Range of Assay (%)	99.8	99.9	99.9

MSD = MesoScaleDiscovery; CV = Coefficient of Variation (precision); LLOD = Lower Limit of Detection (sensitivity); LLOQ = Lower Limit of Quantification (lowest standard); ULOQ = Upper Limit of Quantification (highest standard)

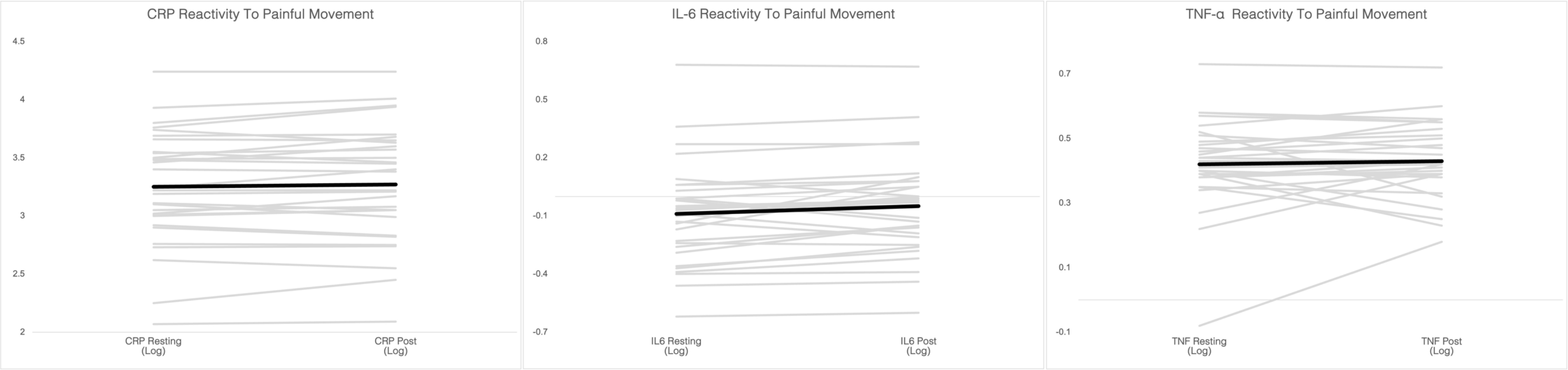

Supplemental Figure 1. Matched Comparisons of Inflammatory Reactivity to Painful Movement

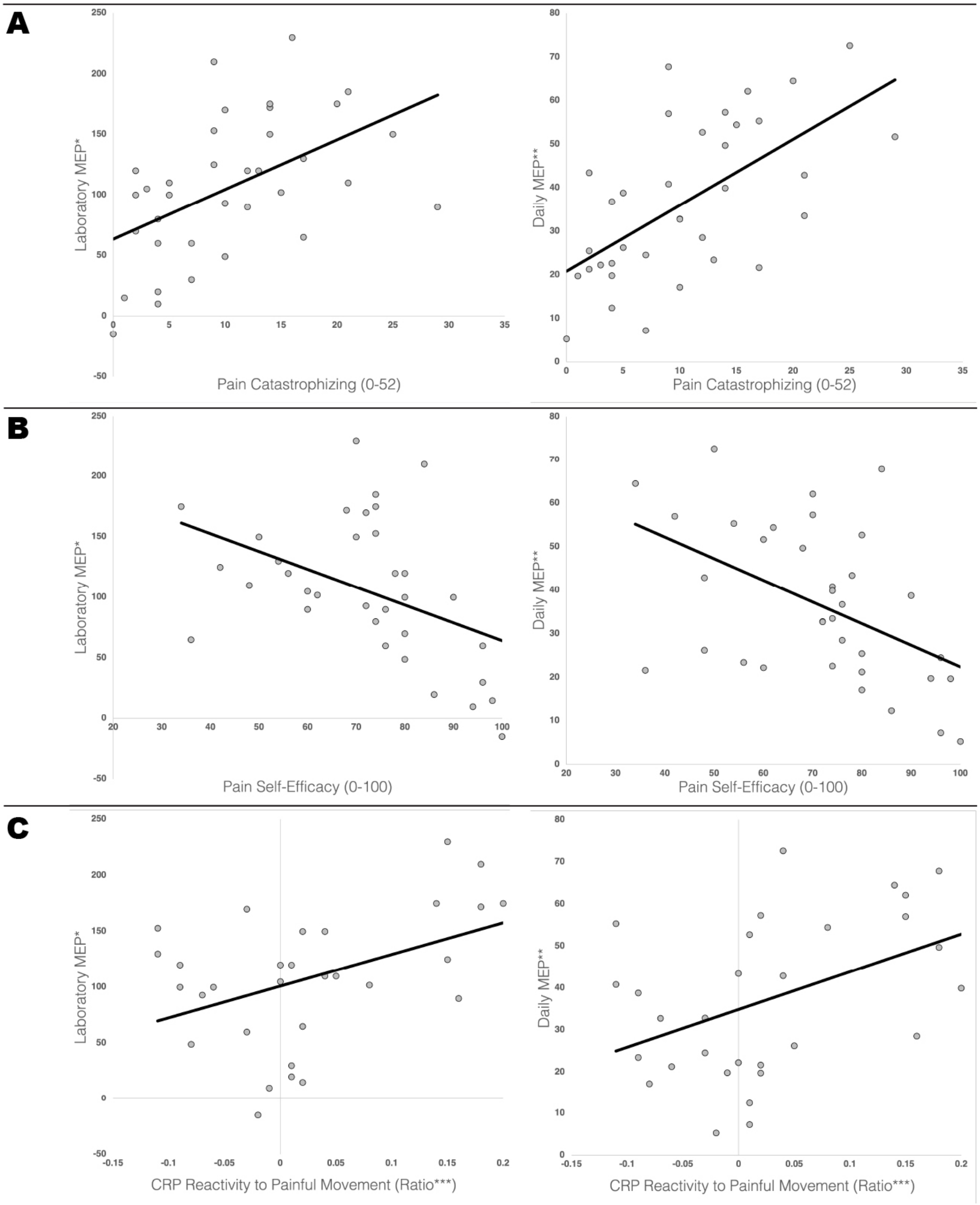

Supplemental Figure 2. Scatterplots of Associations with MEP: Pain Catastrophizing (A), Pain Self-Efficacy (B), CRP Reactivity to Painful Movement (C)

** Aggregate calculation of 4 pain ratings (0 to 100 NPRS) immediately after the 4 physical tasks (0 to 100 NPRS), subtracting pretest resting pain (0 to 100 NPRS).*

*** Average of worst pain (0-100 NPRS) experienced during the movement or physical task considered most painful for each time period of ecological momentary assessment.*

**** Post–pre-test difference in log-transformed values, equating to a normalized ratio*
